# Dataset of Stagnant Water and Wet Surface Label Images for Detection

**DOI:** 10.1016/j.dib.2021.107752

**Published:** 2021-12-23

**Authors:** Sonali Bhutad, Kailas Patil

**Affiliations:** Vishwakarma University, Pune, India

**Keywords:** Stagnant Water, Wet Surface, Water Sanitation, Potential Mosquito Breeding Site Detection, object detection, Computer Vision

## Abstract

Clean water is one of the essential things in life. The running water in natural forms is considered as clean water. To avoid exposure to countless diseases, it is imperative to separate stagnant water from clean water. Thus the main objective of the proposed paper is to create an image dataset of stagnant water and wet surface to detect stagnant water. Accordingly, we considered stagnant water images in different forms and sizes to construct the dataset. In addition to that, brown and black earth surface is considered for the wet surface detection. The dataset consists of 1976 labeled images captured from various angles with annotated files. The dataset images are labelled for two classes, namely water and wet surface. This dataset is highly useful for deep learning experts working in the field of disease control management and post-rainfall earth surface monitoring.

## Specifications Table

**Table:** 

Subject	Computer Vision and Pattern Recognition
Specific subject area	Stagnant Water and Wet Surface Detection
Type of data	Image
How datapoints were acquired	Stagnant water images in different forms such as black, muddy, shiny, and brown were considered for creating the dataset. Images were captured using Samsung Galaxy Note 9 camera with the specifications as below, 12 Mpx, f/1.5-2.4, 26 mm (wide), 1/2.55", 1.4 µm, dual pixel
Data format	Raw Annotated
Parameters for data collection	The dataset is composed of 1976 RGB images (256 × 256 pixels, horizontal 96 dpi, vertical 96 dpi) in JPEG format. All 1976 images are accompanied by annotated (i.e., labeled) image versions that provide membership classes for a significant number of pixels (JPEG format).
Description of data collection	The collection of the image dataset was done in-field, at day-light during varying sunlight. Images represent the top view and side view of stagnant water and wet surface. Annotated images were obtained by manual labeling using the labelImg software.
Data source location	City/Town/Region: Nashik and Mumbai Country: India Latitude and longitude samples/data: 19.9975° N, 73.7898° E, 19.0760° N, 72.8777° E
Data accessibility	Repository name: Dataset of Stagnant Water and Wet Surface with Annotations Data identification number: doi: 10.17632/y6zyrnxbfm.4 Direct URL to data: https://data.mendeley.com/datasets/y6zyrnxbfm/4

## Value of the Data

• This dataset is vital as it contributes to the future applications of the Sustainable Development Goal -6 of the United Nations, i.e., “water and sanitation” (UN SDG -6) [3]. To achieve accuracy in stagnant water detection, we concentrated on the forms of stagnant water rather than the container type, which makes this dataset unique.
• These datapoints are available in the public repository for all the Scientific communities, Research Institutes, Disease control practitioners, and Policymakers.
• According to the best of our knowledge, this is the first dataset that provides annotated stagnant water and wet surface images; that could be used in a wide variety of future research studies related to water and wet surface detection.
• The data may be reused for conducting experiments related to waterlogging area detection, surface water detection, water sanitation, and potential mosquito breeding site detection. Moreover, the researchers who are involved in earth surface detection may benefit indirectly [2].

## 1 Data Description

The dataset folder comprises separate folders for Indoor, Outdoor and Raw data. The Raw data folder images are unprocessed; hence resolution range is given for them (Table 3, Point.5). The images in the other two folders are resized and labelled. The image file name with suffix “s” denotes indoor images. The class file stored in the dataset provides the class name used for labelling. The image count is provided according to the direction of image coverage, location and class for the image samples of the dataset (Table 4).

The dataset contains 1976 annotated images. Each image was stored in JPEG format and accompanied by the txt file's annotation, wherein the annotation labels considered were water and wet surface. In the water image, different forms of water can be seen like muddy, black, shiny, and with reflection. All the images were taken from the top view and the side view. These images were captured during 2019 from two cities, namely Nashik and Mumbai, of Maharashtra, India. Two people were involved in image collection, and each one collected a thousand images, whereas labeling is done manually by one person to avoid annotation mistakes.

Table 1 shows resultant annotations for the labels shown in Fig. 2. The stagnant water and wet surface images with their annotations in the text file are stored in the dataset folder. Based on the image location, the dataset directory structure is divided into two main folders: Indoor and Outdoor. The outdoor folder is divided into three subfolders: a) Water, b) Wet surface, and c) Water and wet surface. Further, these subfolders are divided into two more subfolders: Top view and Side View. The indoor folder consists of Top view folder with water images only (Fig. 3).

**Table 1:** YOLO format annotations.

Class Name	X-min	Y-min	Width	Height
0	0.275391	0.419922	0.347656	0.214844
0	0.048828	0.46875	0.089844	0.25
0	0.197266	0.535156	0.246094	0.09375
0	0.470703	0.388672	0.097656	0.144531
0	0.1875	0.34375	0.125	0.109375
1	0.699219	0.158203	0.59375	0.308594
1	0.765625	0.378906	0.46875	0.148438
1	0.251953	0.089844	0.277344	0.171875
1	0.203125	0.230469	0.398438	0.101562
1	0.166016	0.685547	0.324219	0.183594
1	0.398438	0.607422	0.140625	0.160156
1	0.535156	0.556641	0.125	0.183594

**Fig. 1:**
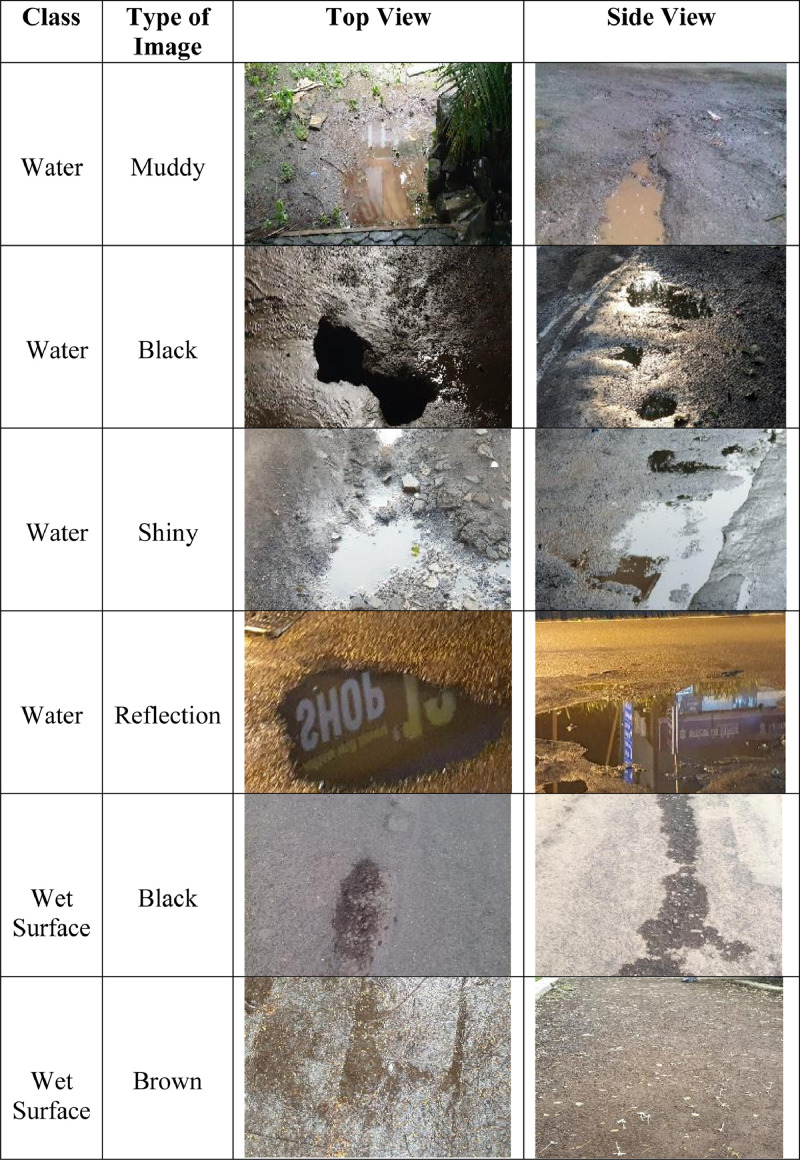
Partial images of the dataset.

**Fig. 2:**
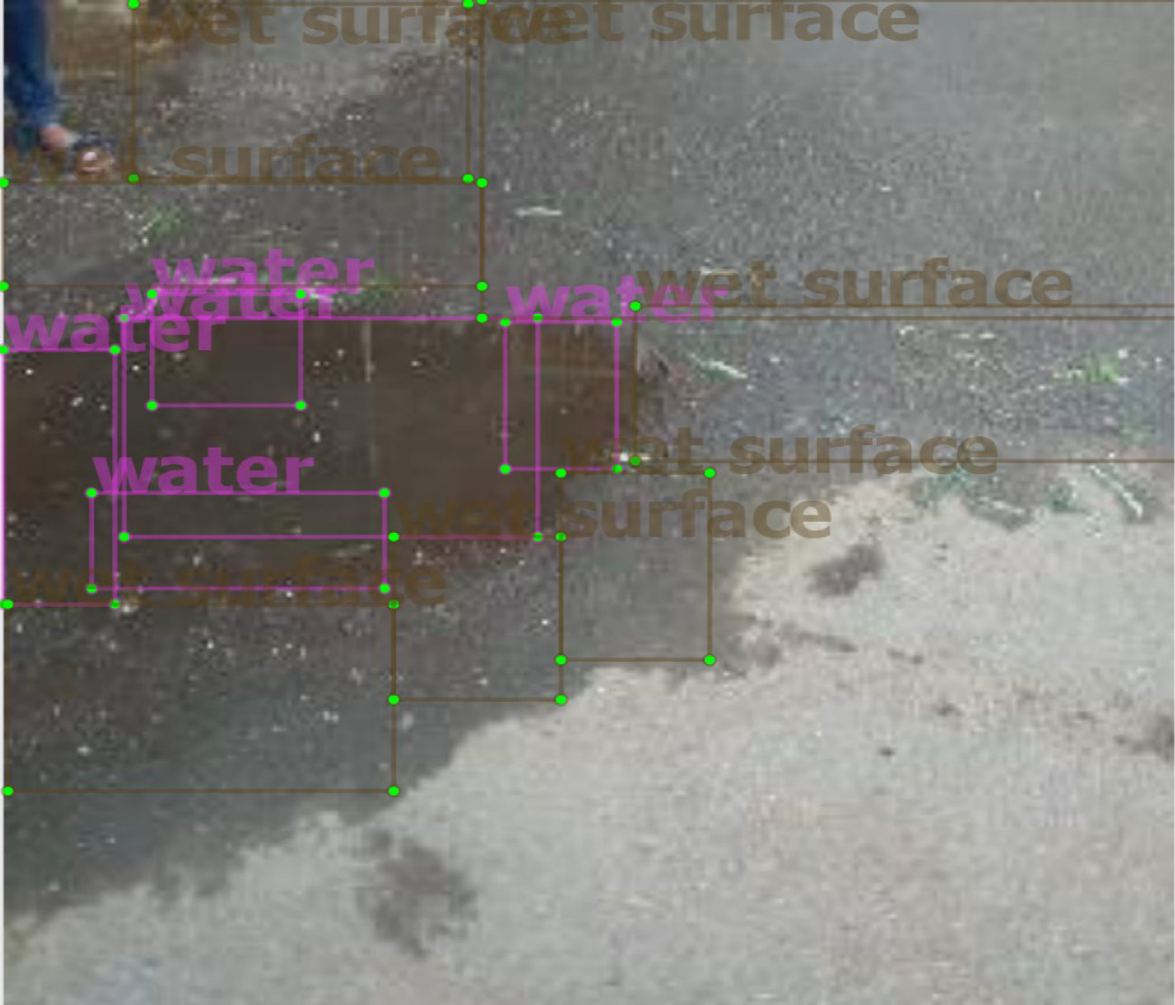
Water and wet surface image with labels (image213.jpeg).

According to photo interpretation, samples are annotated using labelImg software, with a label name associated with their class (Fig. 2). For each image, two classes are used with the names wet surface and water class (Fig. 2). For the wet surface class, brown and black earth surface images were considered as they show resemblance with stagnant water form. While for the water class, stagnant water images in various forms were considered (Fig. 1). To cover the entire water body using rectangle annotations, some labels of the same class may overlap. However, rectangle annotations do not overlap if the class is different [4], [5].

The YOLO format stores the annotations in txt file in the following format,

Class X-min Y-min width height

The values 0 and 1 of the txt file (Table 1) corresponds to the water and wet surface class, respectively (Fig. 2). The following next two values are for the x and y coordinates of the annotation, and the rest two are for the height and width of the annotation.

## 2 Experimental Design, Materials and Methods

### 2.1 Experimental Design

**Fig. 3:**
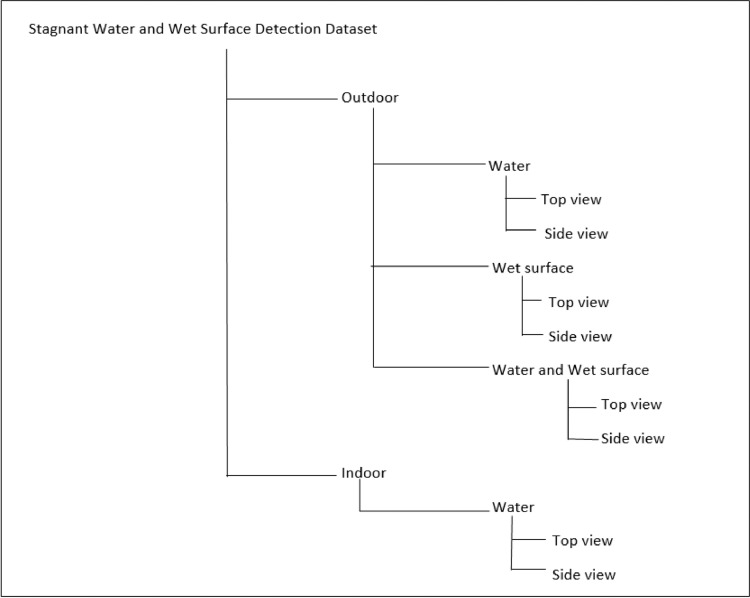
Dataset directory structure.

**Fig. 4:**
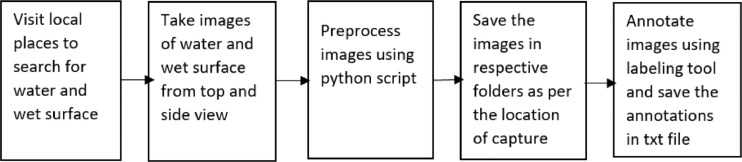
Water and wet surface data acquisition process.

The image data acquisition process is shown in Fig. 4. The water and wet surface images were acquired using Samsung Galaxy Note 9 mobile's high-resolution rear camera. In all 1976, images were captured using a camera and then were segregated and saved in respective folders as per their class and location of capture (Fig. 3). As per [7], we have considered all scenarios while taking images, i.e. indoor and outdoor.

Availability of stagnant water is more during the rainy season, i.e., June and July. Therefore these two months were chosen for capturing images (Table 2).

**Table 2:** Data acquisition requirement.

SI. No.	Year	Month	Frequency	Activity
1	2019	June	Daily	Captured Images in the morning, afternoon, evening, and late evening
2	2019	July	Daily	Captured Images in the morning, afternoon, evening, and late evening

As water reflects the colour of the surroundings, it becomes difficult to detect water surfaces easily in all forms. Hence this dataset was prepared by considering all adverse conditions required for water detection. To cope with adverse conditions, it is necessary to collect images in different forms such as shiny, black, brown, and with reflection. Hence the images were captured in the morning, afternoon, evening, and late evening. Furthermore, to avoid misclassification, wet surface images are also included in this dataset (Table 4).

### 2.2 Materials or Specification of Image Acquisition System

The imaging system consists of a 12 Mpx Samsung Galaxy Note 9 (F2.4, dual pixel) RGB camera, used with a short exposure time within the range of 200– 300 µs (Table 3) [6]. A 15 V battery was used to power all the components of the imaging system. Due to the complexities of terrain, remote sensing data, and classification methods; it is difficult to accurately identify stagnant water [1]. Thus to overcome the weather and time-dependent variations of illumination in an outdoor environment, the dataset consists of stagnant water images in the form of reflections, shiny, muddy, and black water. While capturing images for wet surfaces, images that were similar to stagnant water were considered.

**Table 3:** Specification of image acquisition system.

Sr. No.	Particulars	Details
1	Camera	a) Make and Model: Samsung Galaxy Note 9
		b) Sensor: 12MPx AF sensor
		c) Focus Adjustment: automatic
		d) Lens aperture: F2.4 dual pixel
2	Labelling Software	labelImg
3	Resolution of image	256 × 256 pixels
4	Image Format	JPEG
5	Original Image Resolution Range	Maximum-3264 × 2448 Minimum- 960 × 720

### 2.3 Method

Table 4 describes the classes, number of images taken and the environments in which images are taken. Images were captured at a man's height using a handheld mobile camera for the outdoor scene, while indoor five images of a dish were taken from the top view by bending down. The images were captured for water flakes and puddles available indoors and outdoors. The photos of the wet surface were taken by considering the water retention areas available around the water body and, in some cases, separate wet surface images collected from other locations.

**Table 4:** Water and wet surface dataset details.

Class	Type	Location-wiseImage Count	Direction-wise Image Count	Time of Image Coverage	Count
Water	Shiny, Muddy, Black, With Reflections	Indoor- 5 Outdoor-88	Top view-27 Side view-66	Morning, Afternoon, Evening, and Late Evening	93

Wet surface	Brown, Black	Outdoor- 77	Top view-37 Side view-40	Morning, Afternoon	77

Water and wet surface	Shiny, Muddy, Black, With Reflections, Brown, Black	Outdoor-1806	Top view-302, Side view-1504	Morning, Afternoon, Evening, and Late Evening	1806

## Ethics Statement

This data is available in the public domain, and no funding is received for the present effort. There is no conflict of interest.

## CRediT Author Statement

**Sonali Bhutad**: Methodology, Data Validation, Formal analysis,Writing - OriginalDraft; **Kailas Patil**: Conceptualization, Writing – Review and Editing, Supervision, Project administration.

## Declaration of Competing Interest

The authors declare that they have no known competing financial interests or personal relationships which have or could be perceived to have influenced the work reported in this article.
